# Supplementary material for: Oxyresveratrol Attenuates Inflammation in Human Keratinocyte via Regulating NF-kB Signaling and Ameliorates Eczematous Lesion in DNCB-Induced Dermatitis Mice
Source: Pharmaceutics. 2023 Jun 12;15(6):1709. doi: 10.3390/pharmaceutics15061709 (PMC10300736; doi:10.3390/pharmaceutics15061709)
Supplement: Supplementary file 1 [file pharmaceutics-15-01709-s001.zip › pharmaceutics-2419005-supplementary.pdf]

pharmaceutics

MDPI

Article

Supplementary: Oxyresveratrol Attenuates Inflammation in Human Keratinocyte via Regulating NF-kB Signaling and Ameliorates Eczematous Lesion in DNCB-Induced Dermatitis Mice

Hung Gia Tran, Aussavashai Shuayprom, Patipark Kueanjinda, Asada Leelahavanichkul, Prapai Wongsinkongman, Siriwan Chaisomboonpan, Apiwat Tawatsin, Kriangsak Ruchusatsawat and Jongkonnee Wongpiyabovorn

Supplementary table legends

Table S1. Sequence of primers used in this study.

Supplementary figure legends

Figure S1. Impact of ORV on the viability of cells. HaCat and HEKa cells were exposed to the different concentrations of ORV for 24 h. MTT assay was used to determine cell viability. GraphPad Prism was utilized to calculate the IC_{50} of ORV on each cell type (a). HaCat cell viability was determined using MTT assay after exposed to different concentrations of commercial ORV compare with ORV extraction for 24 h. (b) Commercial ORV purchased from Sigma-Aldrich (St. Louis, MO, USA) was used for comparison on HaCaT.

Figure S2. Cell viability and production of inflammatory cytokines (IL-6 and IL-8) by HaCat cells after treatment with PGN. PGN concentrations were varied in order to find an optimal concentration that was greater than IC50 and still produced a substantial amount of IL-6 and IL-8 cytokines. PGN at 5 µg/ml exerted a modest effect on HaCaT viability as measured by MTT assay (a). Also, PGN at 5 µg/ml can stimulate substantial amount of *IL-6* and *IL-8* expression as measured by PCR (b).

Figure S3. Comparison of anti-inflammatory effect between the ORV compound and commercial ORV from Sigma-Aldrich. The anti-inflammatory effect of ORV was compared with that of a commercial compound from Sigma-Aldrich on *IL-6* (a) and *IL-8* (b). *GAPDH* was used as the internal control. Asterisks indicate statistical significance compared to PGN-stimulated cells. ****, $p < 0.0001$.

Figure S4. Expression levels of genes in HEKa cells after LPS and PGN treatment. PGN and LPS at 10 µg/ml were used to treat HEKa cells and mRNA expression levels of *IL-1β* (a), *IL-6* (b), *IL-8* (c), and *hBD3* (d) were measured. *GAPDH* was used as the internal control. Asterisks indicate statistical significance compared to medium-treated control. **, $p < 0.01$; ***, $p < 0.001$.

Table S1. Sequence of primers used in this study.

Gene	Sequence	T_m (°C)	Length (base pairs)
GAPDH	Fw: 5'-ACC CAC TCC TCC ACC TTT-3'<br>Rv: 5'-CAC CAC CCT GTT GCT GTA G-3'	60	108
IL-1β	Fw: 5'-ACA GAT GAA GTG CTC CTT CCA-3'<br>Rv: 5'-GTC GGA GAT TCG TAG CTG GAT-3'	60	73
IL-6	Fw: 5'-GGC ACT GGC AGA AAA CAA CC-3'<br>Rv: 5'-GCA AGT CTC CTC ATT GAA TCC-3'	60	85
IL-8	Fw: 5'-GAG AGT GAT TGA GAG TGG ACC AC-3'<br>Rv: 5'-CAC AAC CCT CTG CAC CCA GTT T-3'	60	112
hBD3	Fw: 5'-AGC CTA GCA GCT ATG AGG ATC-3'<br>Rv: 5'-CTT CGG CAG CAT TTT GCG CCA-3'	60	206
LL37	Fw: 5'-GCC CAG GTC CTC AGC TAC AAG G-3'<br>Rv: 5'-CTA GGA CTC TGT CCT GGG TAC AAG-3'	60	426

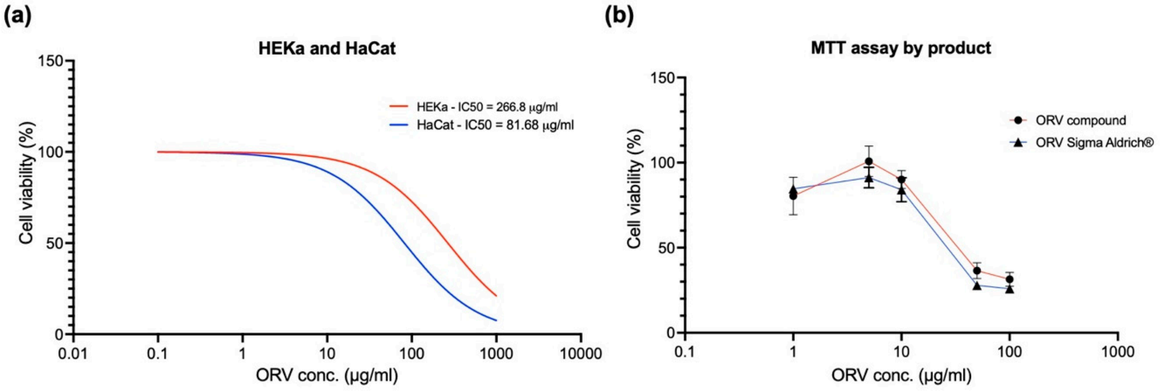

Figure S1. Impact of ORV on the viability of cells. HaCat and HEKa cells were exposed to the different concentrations of ORV for 24 h. MTT assay was used to determine cell viability. GraphPad Prism was utilized to calculate the IC_{50} of ORV on each cell type (a). HaCat cell viability was determined using MTT assay after exposed to different concentrations of commercial ORV compare with ORV extraction for 24 h. (b) Commercial ORV purchased from Sigma-Aldrich (St. Louis, MO, USA) was used for comparison on HaCaT.

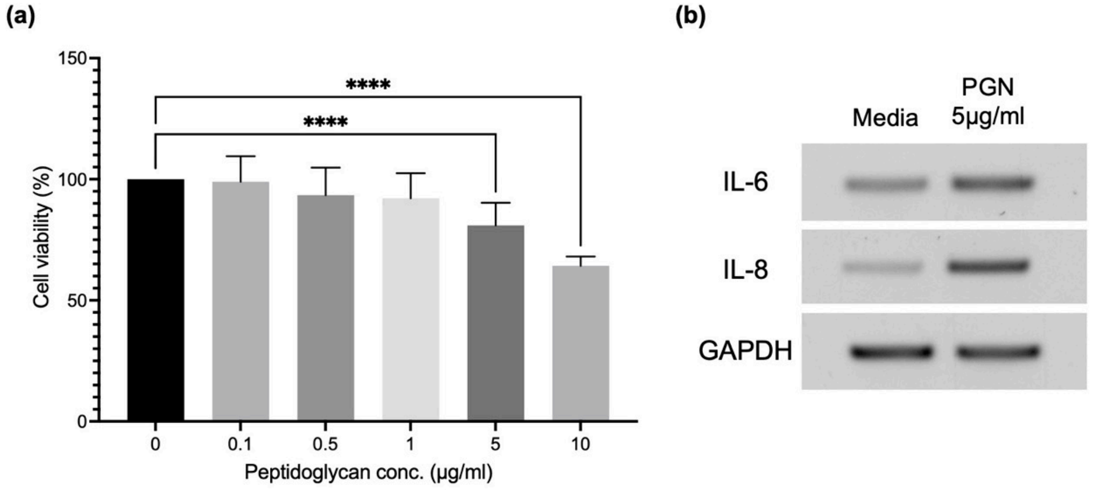

Figure S2. Cell viability and production of inflammatory cytokines (IL-6 and IL-8) by HaCat cells after treatment with PGN. PGN concentrations were varied in order to find an optimal concentration that was greater than IC50 and still produced a substantial amount of IL-6 and IL-8 cytokines. PGN at 5 µg/ml exerted a modest effect on HaCat viability as measured by MTT assay (a). Also, PGN at 5 µg/ml can stimulate substantial amount of *IL-6* and *IL-8* expression as measured by PCR (b).

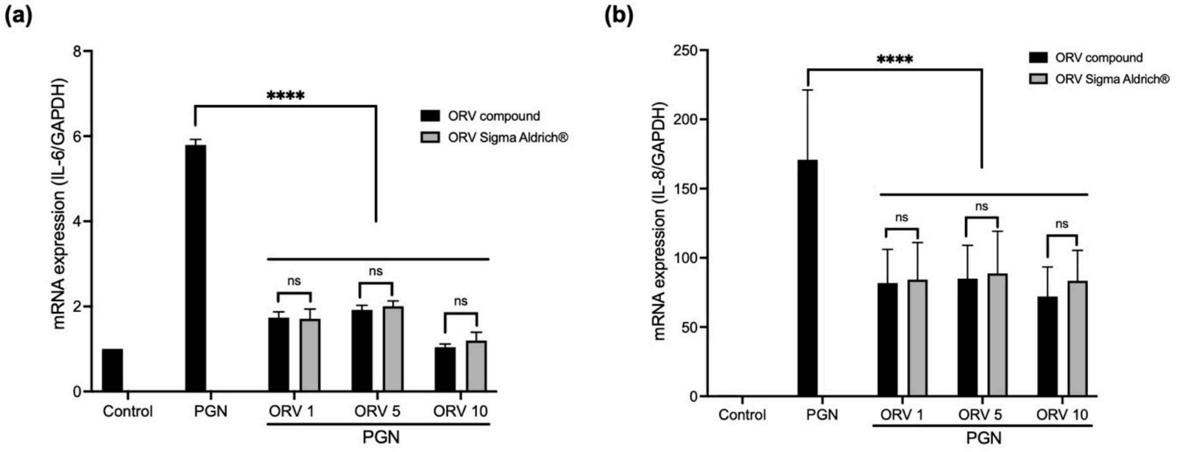

Figure S3. Comparison of anti-inflammatory effect between the ORV compound and commercial ORV from Sigma-Aldrich. The anti-inflammatory effect of ORV on HaCaT was compared with that of a commercial compound from Sigma-Aldrich on *IL-6* (a) and *IL-8* (b). *GAPDH* was used as the internal control. Asterisks indicate statistical significance compared to PGN-stimulated cells. ****, $p < 0.0001$.

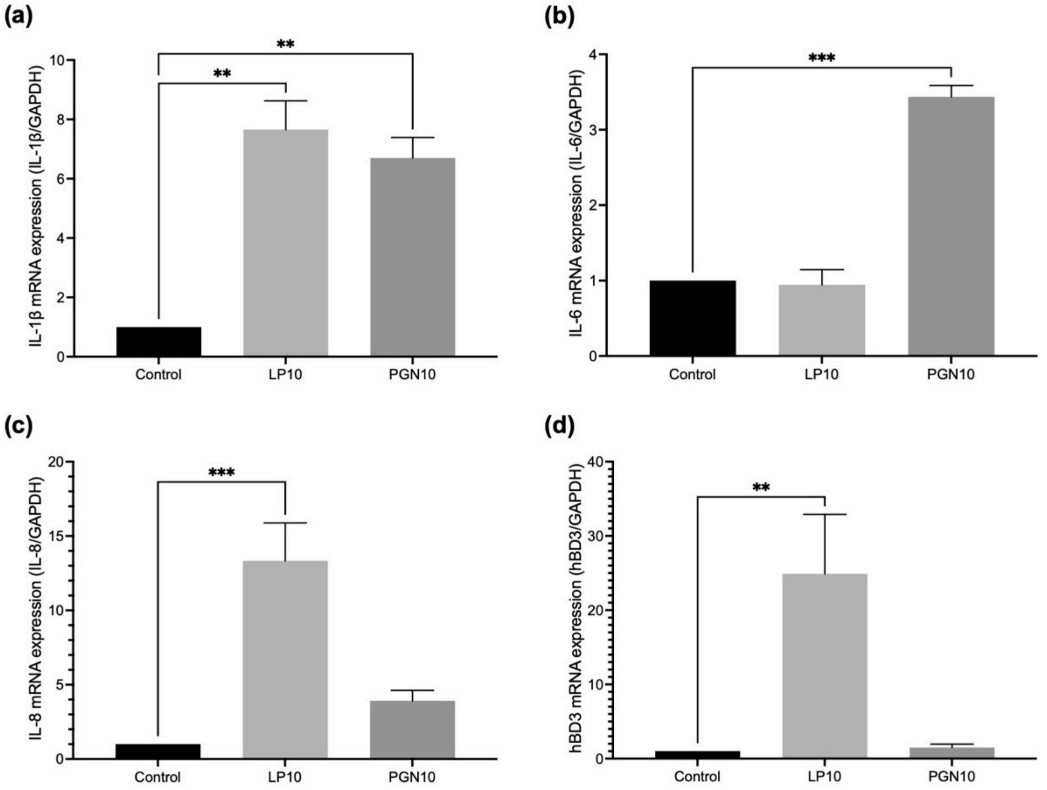

Figure S4. Expression levels of genes in HEKa cells after LPS and PGN treatment. PGN and LPS at 10 μg/ml were used to treat HEKa cells and mRNA expression levels of *IL-1β* (a), *IL-6* (b), *IL-8* (c), and *hBD3* (d) were measured. *GAPDH* was used as the internal control. Asterisks indicate statistical significance compared to medium-treated control. **, $p < 0.01$; ***, $p < 0.001$.
